# Integrated Yoga and Ayurveda therapy for negative symptoms in schizophrenia: A case series highlighting safety and synergistic value of integrative and personalized healthcare

**DOI:** 10.1016/j.jaim.2024.101092

**Published:** 2025-05-23

**Authors:** Kavyashree Kulamarva, Varsha Karanth, Chandrasena Santosh, Chikkanna Umesh, Hemant Bhargav, Venkataram Shivakumar, Kishore Kumar Ramakrishna, Nishitha Jasti, Shivarama Varambally

**Affiliations:** a Department of Integrative Medicine, National Institute of Mental Health and Neurosciences, Bengaluru, India; b Sri Madhusudan Sai Institute of Medical Sciences and Research, Chikkaballapur, Bengaluru, India; c National Institute of Mental Health and Neurosciences, Bangalore, India

**Keywords:** Schizophrenia, Negative symptoms, *Shodhana*, *Shamana*, Ayurveda, Yoga, Integrative

## Abstract

Negative symptoms are a significant contributor to the psychopathology of schizophrenia, primarily affecting socio-occupational functioning. Though effective in managing positive symptoms, most pharmacological agents have limited effects on the negative symptoms of schizophrenia, especially the primary negative symptoms. We discuss five cases (4 males, one female, age range: 24–36 years) of chronic schizophrenia (diagnosed as per ICD-10 criteria) with duration of illness ranging from 2 to 18 years. All the cases had negative symptoms with moderate to extreme severity on the scale for assessment of negative symptoms (SANS) (scores 49 or above; range 49–82). Subjects underwent integrated yoga and ayurveda (IYA) intervention for 12 weeks. Ayurveda intervention included systematic detox *(Panchakarma Shodhana)* and pacifying herbs *(Shamana),* whereas yoga involved the practice of a previously validated yoga module. For all the subjects, anti-psychotic medications were stable for the last eight weeks and did not change during the 12-week IYA intervention and eight weeks after. Post-intervention SANS scores were reduced in all the subjects. The reduction of SANS scores ranged from −15 to −43 (average: −35.6), corresponding to CGI-I ratings of ‘much improved’ in three subjects and ‘minimally improved’ in two subjects. Clinical assessments by an independent psychiatrist at baseline, during IYA (4 weeks), immediately post-IYA (12 weeks), and eight weeks post-IYA did not reveal any new potential side effects due to IYA or worsening of other clinical symptoms or exaggeration of side effects of antipsychotics. This needs further scientific exploration through systematic clinical and mechanistic trials in the future.

## 1 Introduction

Negative symptoms are a core component of schizophrenia, a complex and debilitating mental disorder that affects a person's thoughts, emotions, behavior, and cognition. These symptoms are characterized by deficits or impairments in emotional and behavioral functioning, often leading to a diminished quality of life and socio-occupational functioning [1]. Schizophrenia with predominantly negative symptoms is characterized by five core constructs, i.e., blunted affect, alogia, avolition, asociality, and anhedonia. Currently, available pharmacological agents have limited effects on negative symptoms of schizophrenia [2].

This case series presents a successful integrative (Ayurveda and Yoga) approach to managing chronic schizophrenia with predominantly negative symptoms who showed minimal response to antipsychotics. By highlighting the potential role of an integrative approach in managing negative symptoms of schizophrenia, we aim to provide a new direction to clinicians involved in caring for patients with schizophrenia.

## 2 Patients’ information and clinical findings

Case 1 (RJ) A 35-year-old male from Canada with 20 years' history of tobacco and cannabis use presented with 11 years' history of hearing voices in isolation, ideas of being attacked and targeted by close relatives, reduced sleep, and functional disturbances due to lack of motivation, interest, and brain fogging. He had been diagnosed with paranoid schizophrenia with a partial response to Risperidone 4 mg. The patient refused further increase in the dose of risperidone given the side effects (weight gain and daytime drowsiness). He continued to hear voices in isolation and complained of irritability and poor motivation to interact or work. He smoked 4–5 cigarettes daily and intensely craved nicotine. There were no symptoms suggestive of pervasive low mood or current cannabis use. A family history suggestive of psychotic illness in a 2nd-degree relative was present. Mental status examination revealed poor eye contact, flattened affect (Unchanged facial expression), 2nd person auditory hallucinations, and partial insight.

Case 2 (N) A 36-year-old male presenting with a 3-year history of smiling to himself and talking to himself, diagnosed with undifferentiated schizophrenia with partial response to antipsychotic treatment. He visited for seeking integrative management for poor self-care, decreased social interaction, disinterest in activities, and sleep disturbances with a treatment history of multiple failed trial of various antipsychotics. The patient was previously prescribed clozapine 50 mg, but due to excessive daytime sedation, he had discontinued the medicine. Pre-morbidly, he had anxious/ avoidant traits, but there was no significant family history of psychiatric illness. On mental status examination, he had inappropriate smile. poor eye contact, minimal spontaneous speech (Poverty of Content of Speech), and blunt affect (Unchanged facial expression) but denied any perceptual abnormalities. His cognitive functions were grossly intact, but his judgment and insight were impaired.

Case 3 (RK) A 34-year-old male patient diagnosed with undifferentiated schizophrenia for 18 years visited for seeking integrative treatment given the continuous course of the illness characterized by ideas of persecution, hearing multiple voices in isolation, wandering, lack of initiation (Physical Anergia), poor social interaction (Poor relationships with friends and pears), lack of expressions (Affective non-responsivity), decreased sleep and self-care (Grooming and hygiene). There was a history suggestive of depression and psychosis in the family, but he was reported to have been well-adjusted pre-morbidly. Treatment history revealed multiple failed trials of antipsychotics, and later, he was put on clozapine 200 mg and Amisulpride 300 mg. Over two years, improvement was noticed in his positive symptoms, but he remained dull, lethargic with increased daytime sleepiness and extrapyramidal symptoms. On mental status examination, he had poor eye contact, low speech volume and tone, poor thought content, and blunted affect. His cognitive functions were grossly preserved with partial insight.

Case 4 (GA) A 24-year-old female diagnosed to be suffering from schizophrenia, with an illness duration of 11 years, fluctuating course with a history of emotional abuse, came seeking integrative care for hearing multiple voices in isolation, unprovoked spells of laughing, crying or screaming, and anger outbursts associated with social withdrawal, lack of motivation, indifference to the surroundings, lack of expressions along with sleep disturbances. She was a known case of hypothyroidism. Pre-morbidly, she was reported to have been well adjusted, and on physical examination, she was found to be obese (135.4 Kg). On mental status examination, she had poverty of content of thought and blunted affect (Unchanged facial expression), impaired judgment, and poor insight. Treatment history revealed multiple inadequate trials of antipsychotic drugs, including Amisulpride, Risperidone, Olanzapine, Penfluridol, and Haloperidol. With these drugs, mild to moderate response regarding reduced positive symptoms was noticed, but significant weight gain and menstrual disturbances were seen. Later, the patient was started on Aripiprazole 45mg, with which the patient had some improvement, but there was no change in her negative symptoms.

Case 5 (P) A 24-year-old male, farmer by profession with a history of irrelevant speech, frequent fights with others, aggression, hearing of voices threatening him, suspiciousness, and persecutory ideas along with repetitive, intrusive thoughts with sexual content, social withdrawal, lack of motivation to do his work and impaired self-care for two years was treated with Olanzapine 15 mg and was in remission for a year. Due to weight gain, the patient discontinued the medication, following which there was a relapse of the symptoms for which the medicines were changed to Risperidone 6 mg and Fluoxetine up to 60 mg. Although the patient improved initially, because of weight gain, there was poor medication adherence and a symptom relapse. The patient then approached the department for integrative care. There was no family history of psychiatric disorders, and pre-morbidly, he was reported to have been well-adjusted and an extrovert by nature. On mental status examination, his eye-to-eye contact was fleeting, rapport was established with difficulty, and he was restless and distracted. He was staring vacantly (Decreased Spontaneous Movements), and his reaction time was increased (Increased Latency of Response). His speech volume and tempo were raised, with a lack of prosody, non-goal directed, and irrelevant speech. Persecutory delusions and sexual obsessions were elicited. He had severe formal thought disorder in the form of derailment, tangentiality, and poverty of content of thought. He denied perceptual abnormalities of any modality. His cognitive function, judgment, and insight were impaired.

## 3 Diagnosis, assessment and management

The diagnosis was established based on the International Classification of Disorders (ICD) – 10 after a detailed clinical evaluation by an in-house Psychiatrist. Ayurveda evaluation was done by an Ayurveda physician specialized in Ayurveda psychiatry (*Mano vidnyana*). All the cases had negative symptoms with moderate to extreme severity on the scale for assessment of negative symptoms (SANS) (scores 49 or above; range 49–82) [3]. Case 1 and Case 5 were diagnosed with Paranoid Schizophrenia with incomplete remission. Case 2, Case 3, and Case 4 had predominantly negative symptoms and few positive symptoms with incomplete remission. The scale for assessment of Negative Symptoms (SANS) and Scale for assessment of Positive Symptoms (SAPS) were used for assessment [4]. Assessments were performed at baseline, after four weeks of in-patient treatment (at the time of discharge), and a follow-up assessment was done on the 90th day. Upon discharge, patients were instructed to continue standard care, Ayurveda medications, and yoga practice using video recordings.

Ayurveda diagnosis was based on the involvement of dosha [*V*a*ta*/*Pitta*/*Kapha*] as either involvement of a single dosha or more (combination of two or all the doshas). Personalized Ayurveda and yoga treatment protocols were prescribed considering the individual's *prakruti (constitution) and vikr*u*ti (pathological status)*. Details of ICD-10 diagnosis and its appropriate Ayurveda diagnosis, Ayurveda and yoga intervention, and observations after each therapeutic intervention are summarized in Table no 1, Table 2 (A), Table 2 (B), and Table 3 respectively.

**Table no.1:** ICD-10 diagnosis and its appropriate Ayurveda diagnosis -

	Diagnosis - ICD 10	Diagnosis – Ayurveda
Patient 1 (RJ)	Paranoid schizophrenia	*V*a*taja Unmada* with *Kaphanubandha* (psychosis dominated by *vata* and associated with *Kapha*)
Patient 2 (N)	Undifferentiated schizophrenia	*Kaphaja Unmada* (psychosis dominated by *Kapha*)
Patient 3 (RK)	Undifferentiated schizophrenia	*Kaphaja Unmāda* (psychosis dominated by *Kapha*)
Patient 4 (GA)	Undifferentiated schizophrenia	*Kaphaja Unmāda* with *Vātanubandha* (psychosis dominated by *kapha* and associated with *vata*)
Patient 5 (P)	Paranoid schizophrenia	*Vataja Unmada* with *Kaphanubandha* (psychosis dominated by *vata* and associated with *Kapha*)

**Table 2 (A):** Personalized Ayurveda Intervention (Bio-purificatory) and Yoga measures.

Panchakarma procedure	Patient 1 (RJ)	Patient 2 (N)	Patient 3 (RK)	Patient 4 (GA)	Patient 5 (P)
*Rukshana* (Drying therapy)		*Udwartana* (*kolakulatthadi* powder)	*Udwartana* (*kolakulatthadi* powder)	*Udwartana* (*kolakulatthadi* powder)	
*Snehapana* (Oral intake of medicated ghee	*Kalyaṇaka ghrita*	*Mahapanchagavya ghrita*	*Mahapanchagavya ghrita*		*Kalyaṇaka ghrita*
*Virechana* (Therapeutic Purgation)	*Trivṛut lehya* −60 gm	*Trivṛut lehya* – 70 gm and *triphala Kashaya*	*Trivṛut lehya* −50 gm	*Trivṛut lehya* −50 gm	*Gandharvahastadi eraṇḍa taila* - 45ml
*Nasya* (Nasal instillation)	*Aṇu taila,*	*Aṇu taila*			*Aṇu taila*
*Basti* (Medicated enema)			*Mustadi rajayapana kaṣhaya basti* and *Panchagavya ghrita anuvasana basti*		
*Dhupana* (inhalation of herbal smoke)	*Haridradi dhupana*	*Haridradi dhupana*	*Haridradi dhupana*	*Haridradi dhupana*	*Haridradi dhupana*
Yoga module	Warm-up: Forward-backward bends/twisting/side bending*10 rounds on each side	Warm-up: Forward-backward bends/twisting/side bending*10 rounds on each side	Warm-up: Forward-backward bends/twisting/side bending*10 rounds on each side	Warm-up: Forward-backward bends/twisting/side bending*10 rounds on each side	Warm-up: Forward-backward bends/twisting/side bending*10 rounds on each side
	Sun salutations- slow* 8 rounds	Sun salutations- fast* 8 rounds	Sun salutations-fast* 8 rounds	Sun salutations-fast* 8 rounds	Sun salutations- slow* 8 rounds
	*Naḍishuddhi praṇayama* *6 rounds	*Naḍishuddhi praṇayama* *6 rounds	*Naḍishuddhi praṇayama* *6 rounds	*Naḍishuddhi praṇayama* *6 rounds	*Naḍishuddhi praṇayama* *6 rounds
	Slow Bhastrika*20 strokes*2 cycles in 2 minutes	Fast Bhastrika*20 strokes*2 cycles in 1 minute	Fast Bhastrika*20 strokes*2 cycles in 1 minute	Fast Bhastrika*20 strokes*2 cycles in 1 minute	Slow Bhastrika*20 strokes*2 cycles in 2 minutes
	Mind sound resonance: chanting sound A-U_M* 9 rounds and then AAA*9 rounds;	Mind sound resonance: chanting sound A-U_M* 9 rounds and then AAA*9 rounds;	Mind sound resonance: chanting sound A-U_M* 9 rounds and then AAA*9 rounds;	Mind sound resonance: chanting sound A-U_M* 9 rounds and then AAA*9 rounds;	Mind sound resonance: chanting sound A-U_M* 9 rounds and then AAA*9 rounds;

**Table 2 (B):** Add-on ayurveda and conventional pharmacotherapy.

	Ayurveda Medications	Allopathy Medications
Patient 1 (RJ)	*Ashvagandha* (2 gm) + *Sankhapuṣhpi* (2 gm) + *Vacha* (1 gm) 5 gm twice daily and *Kalyaṇaka ghrita* 15 ml BID	Risperidone 4mg
Patient 2 (N)	*Shankhapuṣpi* (2 gm) + *Gokṣhura* (2 gm) + *Sarpagandha* (1 gm) and *Kalyaṇaka ghrita* 15 ml BID	Clozapine 50 mg
Patient 3 (RK)	*Ashvagandha* (2 gm) + *Shankhapuṣhpi* (2 gm) + *Vacha* (2 gm) 5 gm TID and *Mahapanchagavya ghrita*10 ml BID	Clozapine 200mg Amisulpride 300mg
Patient 4 (GA)	*Ashvagandha* (2 gm) + *Sankhapuṣhpi* (2 gm) + *Vacha* (2 gm), *Drakshadi kaṣhayaṃ* 15ml with 45ml water before food and *Mahapanchagavya ghrita*15 ml HS	Aripiprazole 45mg Tab. Metformin 2g/d Tab. Thyroxine 25mcg
Patient 5 (P)	*Sankhapuṣhpi* (2 gm) + *Gokṣhura* (2 gm) + *Sarpagandha* (2 gm) TID and *Kalyaṇaka ghrita* 15ml BD	Risperidone 4mg Fluoxetine 60mg Trihexyphenidyl 2mg

**Table 3:** Observations after each Ayurveda therapeutic intervention.

Major procedures	Patient 1 (RJ)	Patient 2 (N)	Patient 3 (RK)	Patient 4 (GA)	Patient 5 (P)
Rūkṣaṇa (Drying therapy)	NA	Status quo	Reported lightness in the body	Weight reduction (1kg).	NA
*Virecana* process (ghee intake + sudation + purgation)	Improvement in lethargy and drowsiness	Lesser reaction time and more appropriate social responses.	Reduction in daytime sedation	Mild improvement in hallucinatory behavior and social withdrawal	Significant reduction in restlessness and formal thought disorder
*Basti* (medicated enema)	NA	NA	Improved motivation and social interaction	NA	NA
*Nasya* (Nasal instillation)	Reduction in frequency of auditory hallucinations	Improvement in level of motivation	NA	NA	Decrease in auditory hallucinations and sexual obsessions

NA = Not Administered.

## 4 Therapeutic intervention

The brief yoga module prescribed to the patients was extracted from a validated yoga module for schizophrenia [5]. These concise yoga modules emphasized practicing once daily for two weeks under supervision, followed by home practice using video (the Caregiver also joined the patient and was trained in the module).

## 5 Follow-up and outcomes

**Fig. 1:**
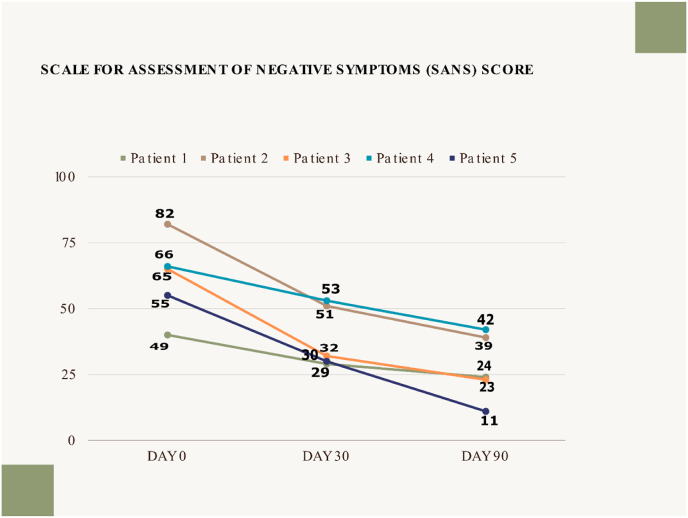
Pre- and post-SANS assessments of patients.

Assessments related to co-morbidities (Hypothyroidism and Extra pyramidal Symptoms) reported by the patients were assessed and they were found under normal limits at the time of admission with ongoing conventional management. The ongoing conventional management for the same was not changed during the IYA treatment. Post-intervention SANS scores were reduced in all the subjects (Fig. 1). The reduction of SANS scores ranged from −15 to - 43 (average: 35.6), corresponding to CGI-I ratings of ‘much improved’ in four subjects (Percentage of improvement – 52.4, - 64.6, - 80 and - 51.2 in Case-1, Case – 3, Case – 4 and Case - 5 respectively) and ‘minimally improved’ in one subject (Percentage of improvement – 36.4 in case −2) [3]. Clinical assessments by an independent psychiatrist at baseline, during IYA (4 weeks), immediately post-IYA (12 weeks), and eight weeks post-IYA did not reveal any new potential side effects due to IYA or worsening of other clinical symptoms or exaggeration of side effects of antipsychotics.

## 6 Discussion

The present case series provides an interesting insight into managing negative symptoms of schizophrenia using a combined approach of modern medicine, Ayurveda, and Yoga. The integration of traditional Indian healthcare approaches with evidence-based modern biomedicine can provide multidimensional care for patients. Fundamental concepts of psychiatric and psychosomatic disorders are explained in Ayurveda under the heading of *Unmāda, which* can be roughly equated to the concept of *psychosis* in modern psychiatry [6]. Given that these patients had associated weight gain and metabolic issues with *Unmada*, *shodhana* (Bio-Purificatory) based therapies targeting the gut were planned for the management. Ayurveda bio-purificatory therapies have been reported to have clinical effects on schizophrenia [6]. Medicated ghee is used for *Snehapana* (oral consumption of medicated ghee) as a pre-requisite to bio-purification, and studies on ghee have shown to have action on the central nervous system in improving learning and memory, in turn enhancing cognition [6,7]. *Virechana* (therapeutic purgation) aids in correcting the metabolism and is effective in metabolic syndrome [8]. Further, studies suggest dysbiosis could exacerbate inflammation in schizophrenia through increased intestinal permeability, and lifestyle changes in schizophrenia have been shown to influence gut microbiome composition [9]. Studies on *Basti* (enema) suggest that enema with herbs can improve the intestinal barrier, regulate gut microbiota dysbiosis, suppress systemic inflammation, and inhibit the overgrowth of conditional pathogenic gut bacteria [10,11]. Applying the paste of herbs over the vertex exerts a calming and relaxing effect and improves stress response by enhancing circulation [12]. Herbs such as *Aśvagandha* (Withania Somnifera), *Sha*n*khapushpi* (Convolvulus Prostrates), *Vaca* (Acorus Calamus Linn), *Gokṣura* (Tribulus Terrestris), *Sarpagandha* (Rauvolfia Serpentina) and *Kalyanaka ghrita* along with bio-purification have shown to be beneficial in managing schizophrenia symptoms [12]. *Mahapanchagavya ghrita* used in the present study has also been clinically effective in negative symptoms and mood disorders [13].

Yoga practices used in these patients were aimed at improving functionality, cognitive deficits, and general well-being. Studies on yoga in subjects with schizophrenia suggest improvement in negative symptoms, emotion recognition deficits, and functioning [14]. Further, a study examining the efficacy of add-on yoga for negative symptoms of schizophrenia found significant improvements in their subjects compared to treatment as usual [2].

Scale for assessment of negative symptoms (SANS) showed an average score of SANS – 53 at baseline, 33 after four weeks of in-patient treatment, and 25 at 12 weeks during follow-up. Overall, the personalized integrative approach substantially changed psychopathology, i.e., a significant drop in negative symptoms was noticed. Patients reported subjective improvement in lethargy, social behavior, and mental and physical activity. At three-month follow-up, patient three could secure a job and had complete remission of the symptoms. Patient 4 could engage in activities like skipping, drawing, and solving maths problems with minimal persuasion. Her eye contact had improved with better spontaneity in response to questions. Patient 5 was noted to be more relaxed and cooperative. He reported reduced auditory hallucinations and sexual obsessions after the course in the hospital. Further, lesser sedation, no daytime sleep, better tolerance towards clozapine dose, and improvement in reaction time in the patients were noticed after the course of integrative treatment. Moreover, the caregiver burden was reduced as per the statements of the patient's relatives.

### 6.1 Limitations and recommendation

All patients could not undergo all the *Shodhana* therapies advised as per Ayurveda texts for managing *unmada* due to the necessity of prolonged inpatient stay to complete the entire course of treatment. Further, each patient was on different antipsychotics, and the dose and duration varied, which could have influenced the outcome. Further, the study did not stratify negative symptoms into primary or secondary categories. As a result, the efficacy of Integrated Yoga and Ayurveda (IYA) cannot be attributed explicitly to primary negative symptoms alone. In the present case series only, SANS was employed to assess the negative symptoms in the patients. We suggest incorporating the latest international guidelines like the European Psychiatric Association (EPA) Guidance on assessing negative symptoms, e.g., persistent negative symptoms (PNS), in clinical settings/research to complement first-generation ones like SANS. It helps in the identification of secondary negative symptoms.

## 7 Conclusion

In the background of limited evidence-based treatment for treating negative symptoms of schizophrenia, an integrated Yoga and Ayurveda approach can be considered a safe and promising line of management to improve patient functioning, quality of life, and productivity with additional benefits of improving their physical health. The current case series of patients of schizophrenia with predominantly negative symptoms have shown clinically relevant improvements through integrative approaches comprising Ayurveda and Yoga as an adjunct. The findings in the current case series can aid in designing future systematic studies to test the efficacy of these integrative approaches in patients of schizophrenia with adverse symptoms in a larger sample.

## Declaration of generative AI in scientific writing

During the preparation of this work, the author(s) have not used any generative AI and AI-assisted technologies in the writing process of the manuscript.

## Declaration of patient's consent

Written informed consent was obtained from the patients to publish this case report, and all identifying information has been removed to protect the patient's privacy.

## Author contributions

Kavyashree Kulamarva (KK), Varsha Karanth (VK), and Santosh C (SC): Data curation, Writing-original draft. Umesh Chikkanna (UC) & Kishore Kumar Ramakrishna (KKR): Conceptualisation, Methodology, Data curation, Writing-review & editing, Supervision. Hemant Bhargav (HB) & Nishitha Jasti (NJ): Conceptualisation, Methodology, Data curation, Writing-original draft, Supervision. Shivakumar Venkatram (SV): Conceptualisation, Methodology, Data curation, Writing-review & editing, Visualization, Supervision. Shivarama Varambally (SSV): Conceptualisation, Writing-review & editing, Supervision.

## Sources of funding

This work was supported by the Centre for Excellence in Research (CoE) grant titled “Yoga and Ayurveda in Neuroscience: Translational Research Accelerator program (YANTRA)” [grant number 3988] under the Ayurswasthya Yojana of Ministry of AYUSH, Government of India.

## Conflict of interest

None.
